# *Portulaca oleracea* L. organic acid extract inhibits persistent methicillin-resistant *Staphylococcus aureus in vitro* and *in vivo*

**DOI:** 10.3389/fmicb.2022.1076154

**Published:** 2023-01-11

**Authors:** Gengsong Liu, Aijing Liu, Cheng Yang, Congcong Zhou, Qiaoyan Zhou, Haizhu Li, Hongchun Yang, Jiahao Mo, Zhidan Zhang, Gonghe Li, Hongbin Si, Changbo Ou

**Affiliations:** ^1^College of Animal Science and Technology, Guangxi University, Nanning, China; ^2^State Key Laboratory of Veterinary Biotechnology, Harbin Veterinary Research Institute, Chinese Academy of Agricultural Sciences, Harbin, China; ^3^Guangxi Zhuang Autonomous Region Engineering Research Center of Veterinary Biologics, Nanning, China; ^4^Guangxi Key Laboratory of Animal Reproduction, Breeding and Disease Control, Nanning, China

**Keywords:** *Portulaca oleracea* L., organic acid, *Staphylococcus* aureus, methicillin-resistant, botanical extract, antibacterial activity

## Abstract

*Staphylococcus aureus* continues to be one of the most important pathogens capable of causing a wide range of infections in different sites of the body in humans and livestock. With the emergence of methicillin-resistant strains and the introduction of strict laws on antibiotic usage in animals, antibiotic replacement therapy has become increasingly popular. Previous studies have shown that *Portulaca oleracea* L. extract exerts a certain degree of bacteriostatic effect, although the active ingredients are unknown. In the present study, the antibacterial activity of the organic acid of *P. oleracea* (OAPO) against *S. aureus* was examined using a series of experiments, including the minimum inhibitory concentration, growth curve, and bacteriostasis curve. *In vitro* antibacterial mechanisms were evaluated based on the integrity and permeability of the cell wall and membrane, scanning electron microscopy, and soluble protein content. A mouse skin wound recovery model was used to verify the antibacterial effects of OAPO on *S. aureus in vivo*. The results showed that OAPO not only improved skin wound recovery but also decreased the bacterial load in skin wounds. Moreover, the number of inflammatory cells and cytokines decreased in the OAPO-treated groups. In summary, this study reports a botanical extract that can inhibit *S. aureus in vitro* and *in vivo*, indicating the potential use of OAPO to prevent and control *S. aureus* infection in the near future.

## 1. Introduction

Staphylococcosis, usually known as *Staphylococcus* infection, is an important zoonotic disease primarily caused by *Staphylococcus aureus*. *S. aureus* is an important clinical pathogen and a common colonizing bacterium found on human skin and mucosal surfaces. It can cause a variety of infections at different sites in humans and animals, ranging from skin and soft tissue infections to serious life-threatening diseases such as osteomyelitis, endocarditis, and necrotizing pneumonia. Moreover, *S. aureus* can pollute food in various ways to produce toxins that cause food poisoning symptoms. Commonly affected foods include milk, raw meat, and frozen food products. In the United States, the number of food poisoning cases caused by *S. aureus* is estimated to be 241,000 per year (Scallan et al., 2011). Many such poisoning events have been reported in China each year (Yan et al., 2012; Lv et al., 2021).

Disinfection and hygienic food/feed management measures are the most important prevention and control strategies for *S. aureus*. *S. aureus*-infected patients and sick animals were first tested for drug sensitivity, and then treated with sensitive drugs. Beta-lactams and glycopeptide antibiotics are used to treat staphylococcosis. However, with the emergence of methicillin-resistant *S. aureus* (MRSA) and the introduction of strict laws on antibiotic usage in animals, an era characterized by the limited use of antibiotics is beginning in China. Many techniques and products replace antibiotics against *Staphylococcus* strains, such as antibacterial peptides, probiotics, prebiotics, enzyme preparations, and traditional Chinese medicine (David and Daum, 2017; Li et al., 2019). Plant extracts are becoming increasingly popular as alternatives to antibiotics, although they cannot completely replace antibiotics. Aqueous extracts of *Galla chinensis* can reduce biofilm formation in methicillin-resistant *S. aureus* and inhibit the invasive ability and pathogenicity of MRSA *in vivo* at a concentration of 31.25 μg/ml (Wu et al., 2019). *Ginkgo biloba* exocarp extracts at concentrations of 4–12 μg/ml were shown to inhibit *S. aureus* and MRSA biofilm formation in a dose-dependent manner and affected biofilm-associated gene expression (Wang et al., 2021). Moreover, herbal extracts and their active constituents can exert synergistic effects in combination with oxacillin or gentamicin against *S. aureus* (Kuok et al., 2017).

*Portulaca oleracea* L. is listed in the National Drug and Food Homologous Catalogue in China with the functions of clearing heat, removing toxins, cooling blood to stop bleeding, and treating dysentery (Zhou et al., 2015). It is mainly used to treat blood dysentery caused by heat toxins, eczema, inflammatory diseases, and redness of the skin. It is a medicine and food homologous drug with a good safety profile in humans. In recent years, many studies have been conducted on its chemical constituents, pharmacological effects, and molecular mechanisms (Rahimi et al., 2019). Two novel amide glycosides isolated from *P. oleracea* exhibit anticholinesterase and scavenging activities (Liu et al., 2021). *P. oleracea* leaf extract also displays antioxidant and anti-inflammatory activities against LPS-induced acute lung injury in a dose-dependent manner (Rahimi et al., 2019). Moreover, the *P. oleracea* extract show relatively potent anti-asthmatic effects owing to decreased nitric oxide production and increased antioxidant markers (Khazdair et al., 2019). Modern pharmacological studies on *P. oleracea* have demonstrated its gastroprotective and hepatoprotective activities in *in vivo* and *in vitro* models (Farkhondeh and Samarghandian, 2019). However, there have been few studies on antimicrobial activity against *S. aureus*. Two unsaturated fatty acids from *P. oleracea* act synergistically with erythromycin against MRSA *in vitro*, possibly by inhibiting methionine sulfoxide reductase (Fung et al., 2017). The carbon dioxide extraction of *P. oleracea* shows clinical effects against significant microorganisms, such as *Escherichia coli* and *S. aureus* (Tleubayeva et al., 2021). However, the chemical constituents that demonstrate antimicrobial activity against *S. aureus* remain unknown.

This study aimed to investigate the anti-*Staphylococcus* activity of an organic acid extract of *P. oleracea in vitro* and *in vivo*. First, the main chemical constituents of *P. oleracea* extract and the anti-*Staphylococcus* effects of the organic acid extract were evaluated using an *in vitro* model. Finally, organic acid extracts were used to cure skin wounds infected with *S. aureus*.

## 2. Materials and methods

### 2.1. Bacterial strain and reagents

Three *S. aureus* strains were used in this study: two field strains (MRSA29 and MRSA85) and one standard strain (ATCC 29213). The 29213 strain was from the First People’s Hospital of Nanning, China. MRSA85 and MRSA29 strains were isolated from clinical samples and stored at Guangxi University (Yang et al., 2020). Trimethoprim-sulfamethoxazole (TMP-SMX) was purchased from the TEYI Pharmaceutical Group (Guangdong, China).

### 2.2. Organic acids of *Portulaca oleracea* preparation

The trunks and leaves of *P. oleracea* were collected from Henan Province (China), and air-dried trunks and leaves were used for organic acid extraction. The obtained *P. oleracea* were identified and stored at the College of Animal Science and Technology, Guangxi University. The organic acid extract was based on a previous study with some modifications (Kim et al., 2016). Briefly, 100 g of dry *P. oleracea* was soaked in a flask with 150 ml petroleum ether overnight and then continuously boiled for 1 h. Finally, the petroleum ether was discarded and dried. The defatted *P. oleracea* was extracted with 95% ethanol at a solid-to-liquid ratio of 1:15 for 1 h at an ultrasonic frequency of 50 kHz. OAPO was obtained by rotary evaporation.

### 2.3. Analysis of the chemical composition of OAPO

Chemical composition analysis of OAPO was performed using an ultra-high-performance liquid chromatography-mass spectrometer (HPLC-MS; Q-Exactive, Thermo). The main chemical compositions of OAPO were identified by comparing its mass spectra with those in a mass spectral library.

### 2.4. Antimicrobial activity of OAPO *in vitro*

#### 2.4.1. Determination of minimum inhibitory concentration

The standard broth microdilution method based on the method from Clinical and Laboratory Standards Institute was used to determine the antibacterial effect of OAPO against *S. aureus* with slight modifications (Zhang et al., 2016). The bacterial suspension [final concentration of 7 × 10^7^ colony-forming units (CFU)/ml] was co-cultured with OAPO at a concentration range of 0.05–50 mg/ml in nutrient broth, with shaking for 16 h at 37°C. The minimum concentration of OAPO that inhibited visible bacterial growth was defined as MIC.

#### 2.4.2. *In vitro* antibacterial activity determined by agar diffusion

The agar diffusion method was used to test the antibacterial properties of OAPO, following a previous study with some modifications (Zhang et al., 2021). The nutrient agar plate was evenly coated with 100 μl *S. aureus* suspension and incubated for 5 min. The sterilized Oxford cup (8 mm in diameter) was placed on the surface of the plate, gently pressed and fixed, and 100 μl solution (experimental group: 25 mg/ml OAPO solution; control group: sterile distilled water) was added into the Oxford cup. The experiment was repeated thrice for each group. Bacterial inhibition zones were observed, and the diameter of each inhibition zone (mm) was measured after incubation at 37°C for 18 h. Based on pharmacological methods, the drug was considered medium sensitive against the bacteria when the diameter was between 10 and 15 mm (Zheng et al., 2022).

#### 2.4.3. Growth and bacteriostasis curves

The MRSA85 strain suspension was co-cultured with a one-fold MIC of OAPO at 37°C at 220 rpm. The OD_600nm_ of the bacterial suspension was determined every 2 h. A growth curve of *S. aureus* was constructed based on the OD_600nm_ value of the corresponding bacterial suspension at the indicated time points (Jiang et al., 2019).

The bacteriostasis curve of OAPO was constructed based on the OD_600nm_ value of the corresponding bacterial suspension at the indicated time points (Wicha et al., 2015). Unlike the growth curve, a one-fold MIC of OAPO was added to the bacterial suspension when the OD_600nm_ value was 1.2. Then a suspension was taken for OD_600nm_ value determination at the indicated time points.

#### 2.4.4. Scanning electron microscopy analysis

The MRSA85 suspension at a concentration of 5 × 10^6^ CFU/ml was added to one-fold or two-fold MIC of OAPO and co-cultured in an incubator at 200 rpm and 37°C for 16 h. MRSA85 without OAPO was used as the control group. The bacterial suspension was centrifuged at 4,000 × *g* for 10 min and washed thrice with 0.1 M phosphate buffer solution (PBS, pH = 7.4). The bacterial precipitate was fixed with 2.5% glutaraldehyde and dehydrated with a gradient concentration of ethanol (50–100%). Finally, the samples were sputtered and plated with gold in an ion-plating machine for 2 min and observed using a scanning electron microscope (Hitachi Regulus 8,100, Japan; Hao et al., 2021).

#### 2.4.5. Cell wall damage assay

The effect of OAPO on the cell wall was determined by alkaline phosphatase (AKP) leakage (Hu et al., 2019). The bacterial sample was preprocessed as described in the SEM analysis. The sample was collected at a specific time point and centrifuged at 4,500 × *g* for 10 min. The supernatant was used to determine the AKP content using an AKP kit (JianCheng, Nanjing, China) following the manufacturer’s instructions.

#### 2.4.6. DNA leakage content induced by OAPO

The bacterial sample was preprocessed as described in the SEM analysis. The obtained MRSA85 bacterial supernatant was used for DNA content determination using a full-wavelength spectrophotometer for nucleic acid detection (Tiangen, China). This was repeated three times, and the DNA content was calculated as the mean ± standard deviation (Hu et al., 2019).

#### 2.4.7. Soluble protein content influenced by OAPO

The bacterial sample was preprocessed as described in the SEM analysis. The obtained MRSA85 bacterial precipitate was completely dispersed in PBS and subjected to ultrasonication. The soluble protein content in the ultrasonic crushing liquid was determined using a BCA protein determination kit and sodium dodecyl sulfate-polyacrylamide gel electrophoresis (SDS-PAGE; Zhang et al., 2017).

### 2.5. Experimental animal grouping and treatment

A murine skin wound infection model was used to validate the *in vivo* efficacy of OAPO against *S. aureus*. The animal study protocols were approved by the Animal Management and Ethics Committee of Guangxi University. Seventy-eight male Kunming mice weighing 18–22 g were housed in a controlled environment at 25°C with a 12 h light–dark cycle and free access to food and water. After the mice were adapted to the environment, they were randomly divided into six groups, with 11 to 14 mice in each group according to treatment: non-infected wound control, infected wound control, infected wound TMP-SMX-treated control, and infected wound OAPO-treated groups.

For dermal wound development, these mice were anesthetized with an intraperitoneal administration of ketamine (25 mg/kg body weight) and xylazine (5 mg/kg body weight) mixture before the surgical procedure (Gupta and Tyagi, 2021). The dorsal surface area of each mouse was shaved and sterilized with 75% ethanol. A full-thickness excision wound of 2 × 2 cm was created. Then the wound was inoculated with 100 μl MRSA85 suspensions at 10^8^ CFU/ml, except for the mice in the non-infected wound control group, which were inoculated with 0.9% physiological saline. To ensure that these wounds were successfully infected with MRSA85, the inoculation treatment was repeated 12 h after the first infection. All the mice were orally administered the corresponding drugs. In the OAPO-treated groups, the mice were orally administered 125, 250, and 500 mg/kg body weight. In the TMP-SMX-treated group, the dosages of TMP and SMX were 320 and 1,600 mg/kg of body weight, respectively, and administered orally twice daily (Guo et al., 2013; Miller et al., 2015). The mice in the non-infected and infected wound control groups were treated with the same volume of water. The mice were monitored daily and weighed throughout the study period of 7 days. The wound area was examined daily. All mice were euthanized by CO_2_ inhalation on the last day of the study, and skin wound tissues were collected for further analyses. Whole blood was collected in tubes for serum analysis.

### 2.6. Skin wound closure rate

At the end of the study, each wound was traced by placing a transparent sheet over it. The traced area was placed over a metric grid, and the number of known area squares was counted to calculate the total wound area. The wound closure rate was calculated according to the formula: (A_0_ - A_8_)/A_0_ × 100%, where A_0_ is the wound area on day 0 of wounding, and A_8_ is the wound area at the end of the study (Gupta and Tyagi, 2021).

### 2.7. Quantification of bacterial load in wound

A piece of skin wound tissue was collected, weighed, and homogenized in 1 ml of PBS. Tissue homogenates (100 μl) were serially diluted and inoculated onto brain-heart infusion agar plates. The plates were then incubated under aerobic conditions at 37°C for 24 h. Viable CFUs were counted and plotted as log_10_ (CFU/g wound tissue; Carneiro et al., 2021).

### 2.8. Histological analysis

Skin wound tissues from the infected mice were excised for histological analysis. Tissues were fixed in formalin and embedded in paraffin. The embedded tissues were sliced into 4.5 μm-thick sections and stained with hematoxylin/eosin (HE). The slides were examined for cellular infiltration, blood coagulation, and presence of bacterial aggregates under a light microscope (Simonetti et al., 2017).

### 2.9. Determination of inflammation cytokines by enzyme-linked immunosorbent assay (ELISA)

The levels of the inflammatory cytokines IL-1β, IL-6, and TNF-α in the serum of each mouse were determined at the end of the study using commercial ELISA kits (Nanjing Boyan, China). Serum samples were analyzed according to the manufacturer’s instructions. The results are expressed as pg./mL of serum.

### 2.10. Statistical analysis

The results are expressed as mean ± standard deviation. Statistical evaluations were performed by analysis of variance using GraphPad Prism 5.0 (GraphPad Software Inc., San Diego, CA, United States). Differences were considered statistically significant at *p* < 0.05.

## 3. Results

### 3.1. Chemical compositions of the organic acid part of *Portulaca oleracea*

**Figure 1:**
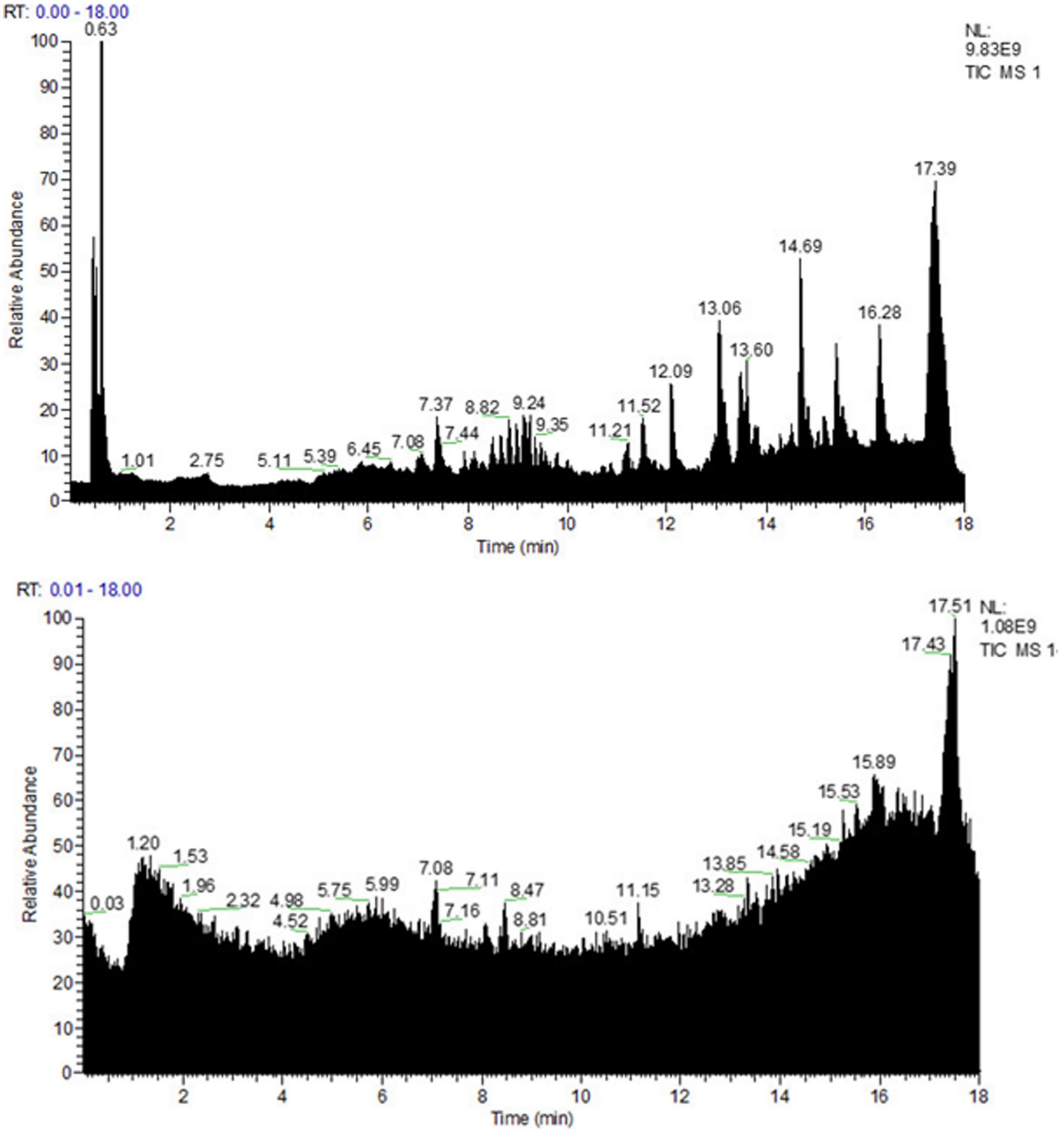
Spectra fingerprint of chemical composition analysis of organic acid of *Portulaca oleracea* L. (OAPO) by ultra-high-performance liquid chromatography-mass spectrometer (HPLC-MS). Upper: positive mode (+); Lower: negative mode (−).

To explore the chemical composition of the organic acid part of *P. oleracea*, HPLC-MS was used to separate and identify the chemical composition of OAPO, and the total ion chromatogram was established, as shown in Figure 1. Moreover, Table 1 shows that the first 25 organic acids in OAPO comprised 5.78% of the total content, including α-eleostearic acid, palmitic acid, l-pyroglutamic acid, linoleic acid, stearidonic acid, azelaic acid, d-pantothenic acid, 6-hydroxypicolinic acid, and phloionolic acid.

**Table 1:** Top 25 organic acids chemicals in OAPO.

Number	Name	Formula	RT [min]	Content (%)
1	α-Eleostearic acid	C18 H30 O2	13.493	1.26142
2	Palmitic acid	C16 H32 O2	11.501	0.99667
3	L-Pyroglutamic acid	C5 H7 N O3	0.641	0.72849
4	9-Oxo-10(E),12(E)-octadecadienoic acid	C18 H30 O3	13.508	0.49083
5	2′-deoxymugineic acid	C12 H20 N2O7	0.604	0.39817
6	N-Benzoylaspartic acid	C11 H11 N O5	6.118	0.22418
7	D-Pantothenic acid	C9 H17 N O5	4.101	0.19323
8	(12Z)-9,10,11-trihydroxyoctadec-12-enoic acid	C18 H34 O5	11.615	0.16167
9	Stearidonic acid	C18 H28 O2	13.141	0.15746
10	Azelaic acid	C9 H16 O4	9.172	0.13633
11	6-Hydroxypicolinic acid	C6 H5 N O3	0.668	0.10979
12	Phloionolic acid	C18 H36 O5	11.528	0.1057
13	3,8,13,17-tetramethyl-12-vinyl-2,7,18-Porphinetripropionic acid	C35 H36 N4O6	14.828	0.10238
14	(2S)-3-Phenyl-2-({[(3S,4S,5R)-2,3,4-trihydroxy-5-(hydroxymethyl)tetrahydro-2-furanyl]methyl}amino)propanoic acid	C15 H21 N O7	0.63	0.09536
15	10,16-Dihydroxyhexadecanoic acid	C16 H32 O4	11.773	0.0903
16	trans-3-Indoleacrylic acid	C11 H9 N O2	5.09	0.08367
17	4-Guanidinobutyric acid	C5 H11 N3 O2	0.61	0.08359
18	(2R)-2-[(2R,5S)-5-[(2S)-2-hydroxybutyl]oxolan-2-yl]propanoic acid	C11 H20 O4	9.172	0.07039
19	3-[14-Ethyl-13-formyl-21-(methoxycarbonyl)-4,8,18-trimethyl-20-oxo-9-vinyl-3-phorbinyl]propanoic acid	C35 H34 N4O6	14.832	0.06261
20	12-oxo phytodienoic acid	C18 H28 O3	11.737	0.05786
21	3-oxopalmitic acid	C16 H30 O3	13.255	0.05415
22	Ferulic acid	C10 H10 O4	7.85	0.04826
23	4-Isobutylbenzoic acid	C11 H14 O2	7.931	0.03314
24	3-Phenylpropanoic acid	C9 H10 O2	0.633	0.02632
25	2-Oxobutyric acid	C4 H6 O3	0.592	0.00796

### 3.2. OAPO inhibits *Staphylococcus aureus* replication *in vitro*

The MIC of OAPO was 12.5 mg/ml, as determined by the broth microdilution method. Moreover, the Oxford cup test showed that the diameter of the inhibition zone of OAPO was 13.5 ± 0.5 cm at a concentration of 25 mg/ml, which indicated that OAPO was medium sensitive against MRSA85.

**Figure 2:**
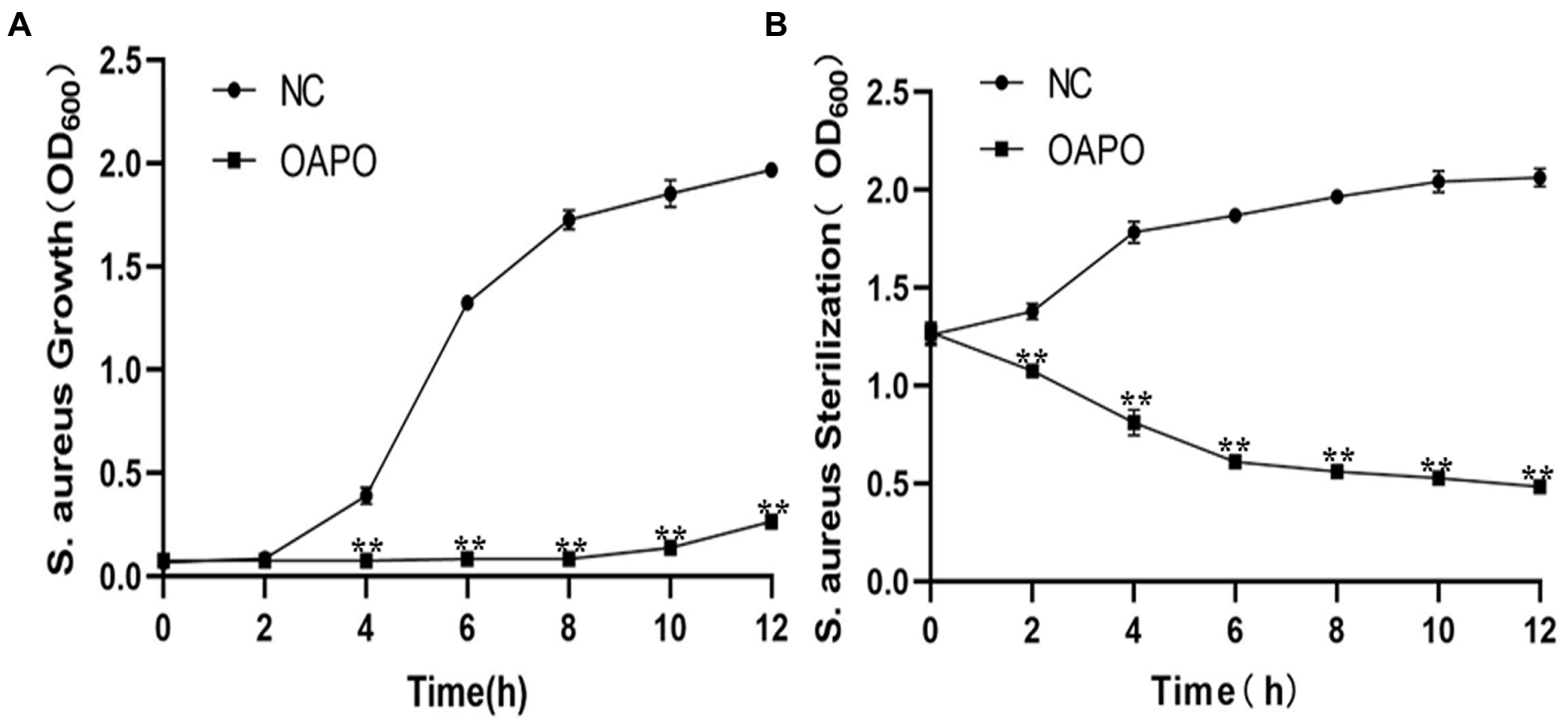
Organic acid of *Portulaca oleracea* L. (OAPO) killing of *Staphylococcus aureus* visualized by the growth **(A)** and bacteriostasis curves **(B)**. The OD_600nm_ of *S. aureus* culture suspension at 0, 2, 4, 6, 8, 10, and 12 h post-coculture with or without 1-fold minimum inhibitory concentration (MIC) of OAPO. NC: negative control, *S. aureus* culture without OAPO; OAPO: *S. aureus* culture with 1-fold MIC of OAPO. **indicates *p* < 0.01 when compared with the negative control group (NC, untreated with OAPO).

OAPO toxicity against *S. aureus* was determined by measuring staphylococcal growth with a 1-fold MIC of OAPO (Figure 2A). The results showed that the OD_600nm_ measured for OAPO-treated bacteria decreased over the course of the experiment, indicating that OAPO is bactericidal to *S. aureus*. OAPO inhibited the growth of *S. aureus* and prolonged the time required to stabilize *S. aureus*. A bacteriostatic curve was constructed to determine whether OAPO was bactericidal or bacteriostatic (Figure 2B). Combined with the one-fold MIC of OAPO, the absorbance value of *S. aureus* steadily declined from the beginning of the experiment.

### 3.3. Antibacterial mechanism of OAPO against *Staphylococcus aureus*

#### 3.3.1. Destructive effects of OAPO on the integrity of the bacterial cell wall and membrane

**Figure 3:**
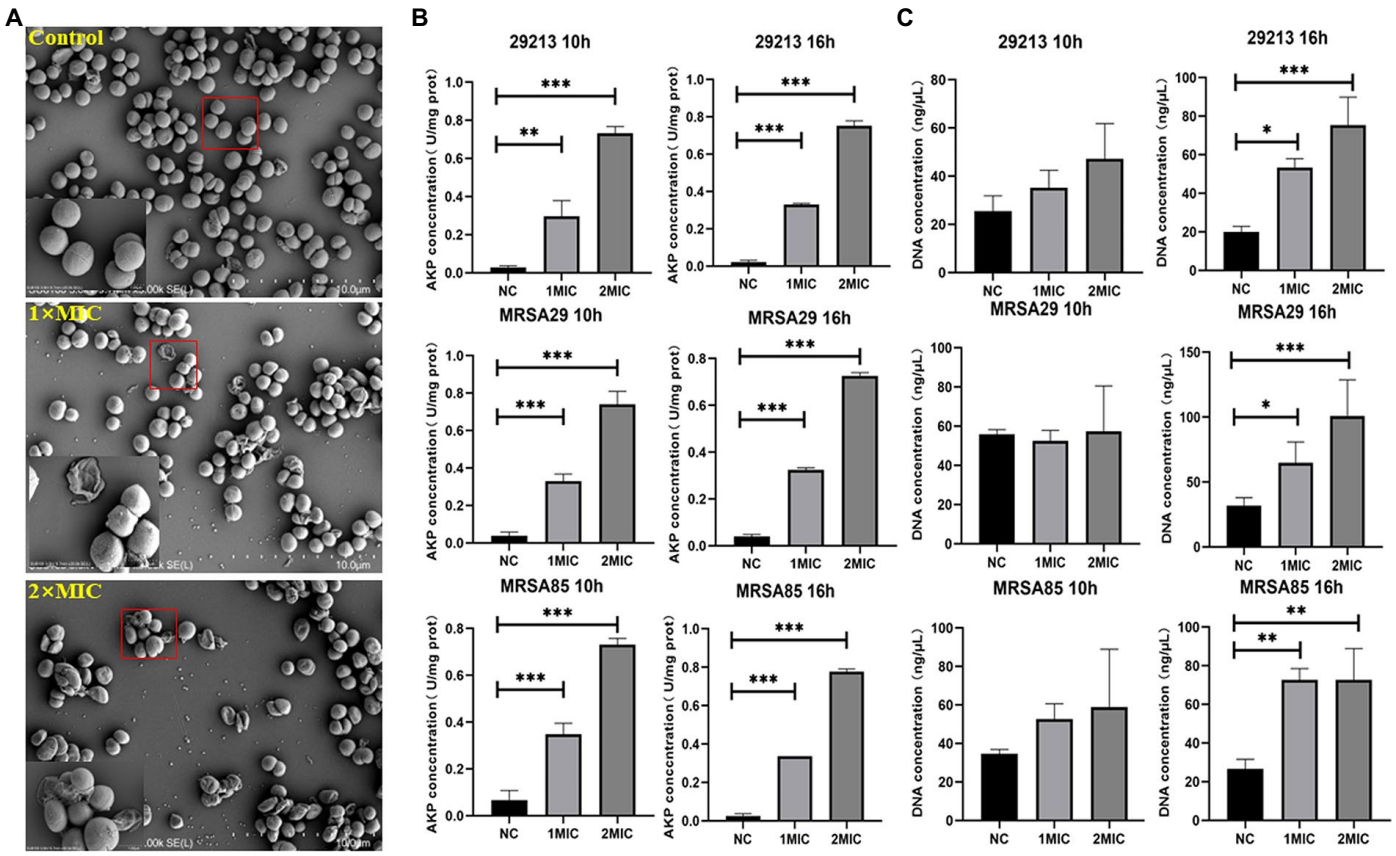
Antibacterial effects of organic acid of *Portulaca oleracea* L. (OAPO) on the morphology and integrity of the cell wall and membrane of *Staphylococcus. aureus*. **(A)** The morphology changes of *S. aureus* untreated (Control) or treated with 1× and 2× minimum inhibitory concentration (MIC) of OAPO by scanning electron microscopy (SEM). A red square indicates that this part was magnified by 10 times. **(B)** Effect of OAPO on the cell wall of *S. aureus* (29,213, MRSA29, and MRSA85 strains) at 10 and 16 h post-treatment with one-fold and two-fold MIC of OAPO (or after no treatment). The alkaline phosphatase (AKP) concentration of the bacterial culture solution was determined. **(C)** Effect of OAPO on the integrity of the cell membrane of *S. aureus* (29,213, MRSA29, and MRSA85 strains) at 10 and 16 h post-treatment with one-fold and two-fold MIC of OAPO (or after no treatment). The DNA concentration of the bacterial culture solution was determined. *indicates *p* < 0.05, **indicates *p* < 0.01, ***indicates *p* < 0.001 when compared with the negative control group (NC, untreated with OAPO).

The SEM images of the morphological changes induced by OAPO are shown in Figure 3A. In the untreated *S. aureus* control group, almost all bacteria showed a normal and intact morphology with a plump surface; in other words, they were uniform in size and shape. However, OAPO-treated bacteria showed noticeable morphological changes. One-fold MIC of OAPO induced considerable morphological changes with damaged cell walls, shriveled cells, and irregular shapes. In the two-fold MIC-OAPO group, the morphologies were more damaging.

AKP, an enzyme between the cell wall and the cell membrane, is undetectable when the morphology of the bacteria is intact. Owing to increased permeability, the AKP content in the bacterial culture solution was positively correlated with the degree of cell wall destruction. Figure 3B shows the AKP concentration in the bacterial culture solution post-administration of OAPO. OAPO significantly increased the AKP content in the culture solutions of 29,213, MRSA29, and MRSA85 strains (*p* < 0.01) compared to that in the untreated control group. Moreover, the effects of OAPO on AKP content were dose-dependent.

The amount of nucleic acid leakage in the bacteria treated with OAPO was also measured to verify the effects of OAPO on the permeability of the bacterial cell membrane. As shown in Figure 3C, the DNA concentration in the bacterial culture solution was not affected 10 h post-treatment with OAPO. However, OAPO significantly increased the DNA concentration 16 h post-administration in all three bacterial strains: 29213, MRSA29, and MRSA85.

#### 3.3.2. Effects of OAPO on soluble protein content

**Figure 4:**
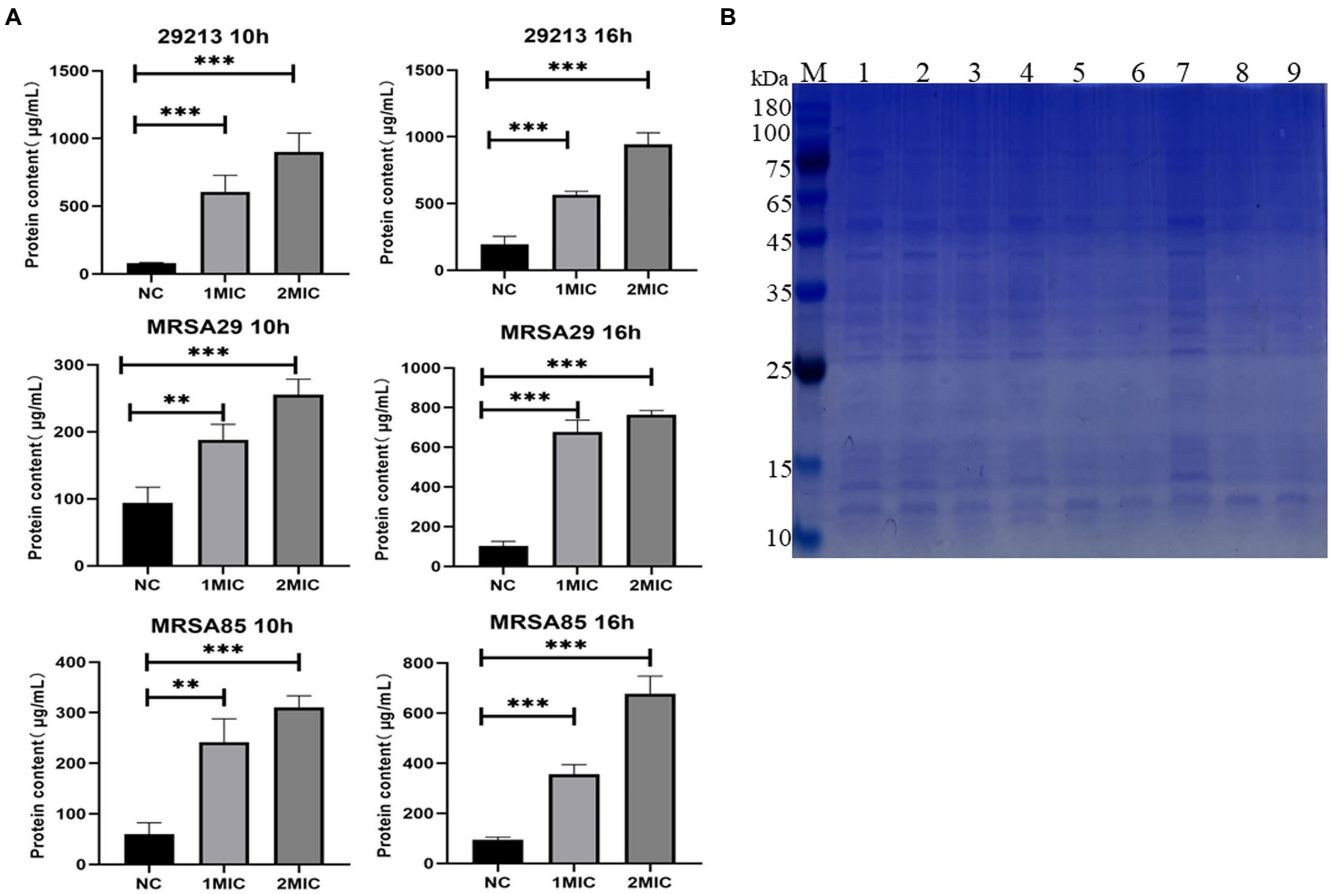
Organic acid of *Portulaca oleracea* L. (OAPO) increases the soluble protein content of bacterial culture solution. **(A)** The soluble protein content of *Staphylococcus aureus* (29,213, MRSA29, and MRSA85) at 10 and 16 h post-treatment with 1× and 2× minimum inhibitory concentration (MIC) of OAPO was determined by the BCA kit. *indicates *p* < 0.05, **indicates *p* < 0.01, ***indicates *p* < 0.001 when compared with the negative control group (NC, untreated with OAPO). **(B)** Sodium dodecyl sulfate-polyacrylamide gel electrophoresis (SDS-PAGE) of bacterial cells treated with OAPO. M: Marker, Lane 1/4/7: 29213, Lane 2/5/8: MRSA29, Lane 3/6/9: MRSA85; Lane 1/2/3: treated with two-fold MIC of OAPO, Lane 4/5/6: treated with one-fold MIC of OAPO, Lane 7/8/9: untreated with OAPO.

The results of the analysis using the BCA protein concentration determination kit and SDS-PAGE profiles of the bacterial proteins are shown in Figure 4. Compared to the those in the untreated control group at the same culture time, the soluble protein contents of 29,213, MRSA29, and MRSA85 were significantly increased at 10 and 16 h post-OAPO treatment (*p* < 0.01), respectively. As the concentration of OAPO and treatment duration increased, the effect of OAPO on the soluble protein content became more significant.

### 3.4. OAPO improved *Staphylococcus aureus*-infected skin injury recovery

**Figure 5:**
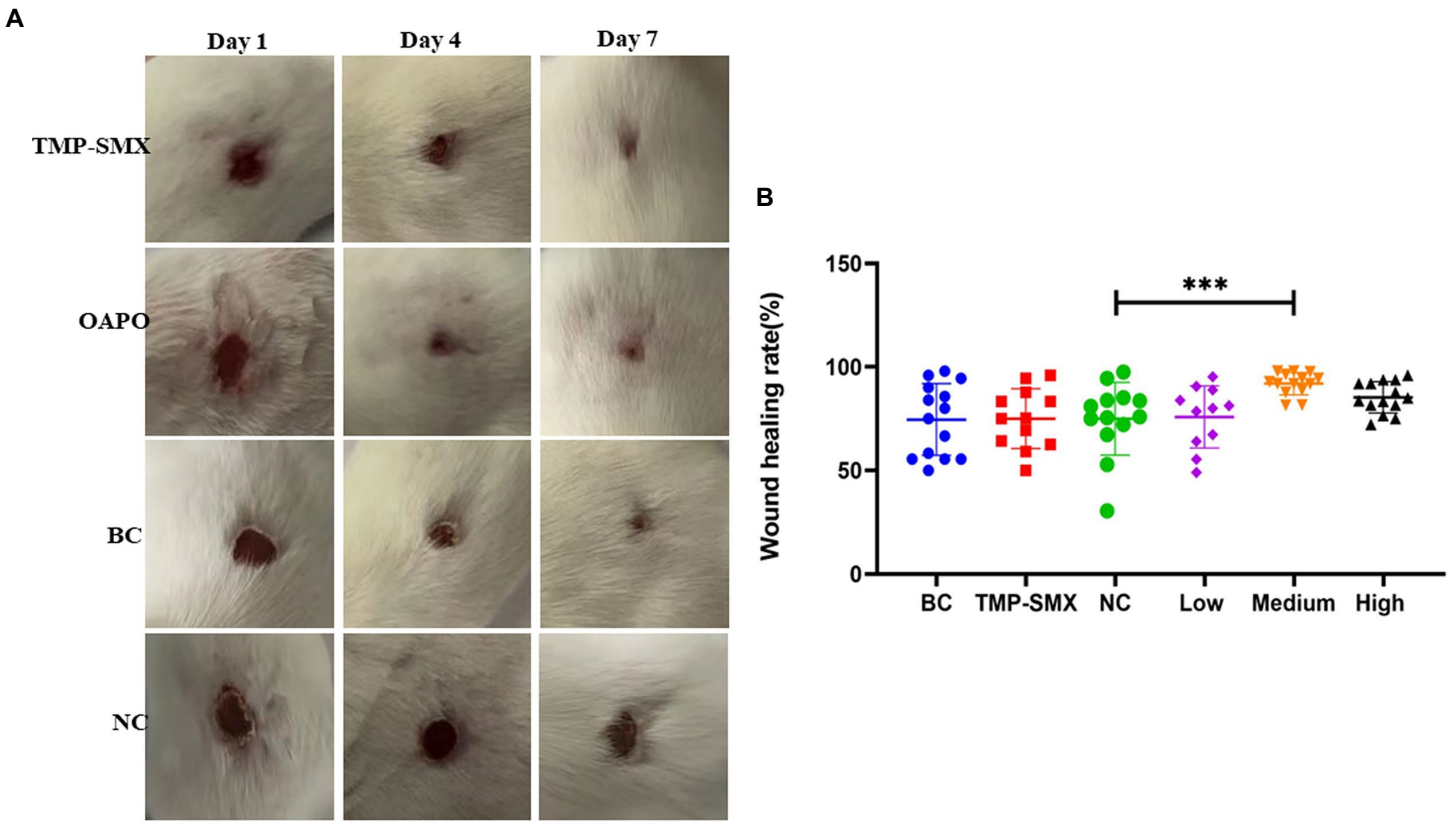
Effects of organic acid of *Portulaca oleracea* L. (OAPO) on skin wound recovery. **(A)** Skin wound healing appearance of mice on days 1, 4, and 7 post-inoculation with *Staphylococcus aureus* MRSA85. TMP-SMX: trimethoprim-sulfamethoxazole; OAPO: OAPO medium group; BC: blank group, uninfected, no drug; NC: infected without drug. **(B)** Scatter diagram of the healing rate of mice on day 7 post-inoculation with *S. aureus* MRSA85. ***indicates *p* < 0.001 when compared with the negative control group (NC, infected without drug).

To explore the effects of OAPO on skin injury induced by *S. aureus* infection, a mouse dermal wound model was used for macroscopic analysis (Figure 5). In the infected control group, slough formation in the wound bed started on day 1 and began to cover the wound thickly on subsequent days. In the non-infected control group, the wound bed started to dry around the edges on day 1, and the wound was almost completely healed at the end of the experiment. In the OAPO- and TMP-SMX-treated infected groups, the wound bed area became smaller than that of the infected control group from days 4 to 7 (Figure 5A). Moreover, the wound healing rate was calculated on day 7, and the results showed that the medium dosage of OAPO significantly increased the wound healing rate compared with that in the infection control group (*p* < 0.01). Interestingly, the wound healing rate of all mice in the medium dosage OAPO group was more than 80% (Figure 5B).

### 3.5. OAPO decreased bacterial load and inflammation cytokines in the skin wound

**Figure 6:**
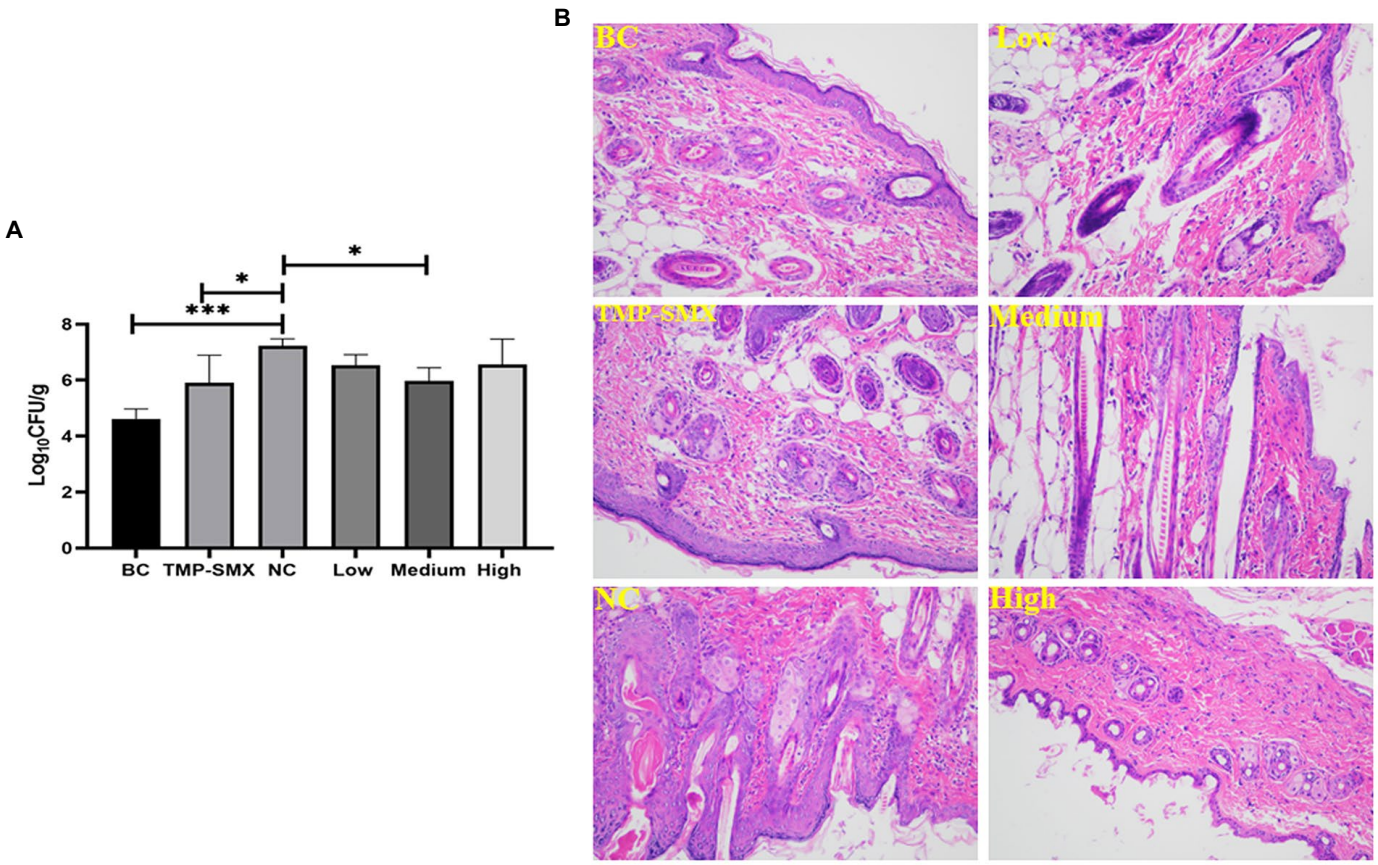
Effects of organic acid of *Portulaca oleracea* L. (OAPO) on skin wound bacterial load **(A)** and histopathology changes **(B)** on day 7 post-inoculation with *Staphylococcus aureus* MRSA85. *indicates *p* < 0.05, ***indicates *p* < 0.001. TMP-SMX: trimethoprim-sulfamethoxazole; BC: blank group, uninfected, no drug; NC: infected without the drug; low, medium and high means groups are given low, medium, and high dosages of OAPO, respectively.

The bacterial load in the wound tissue was assessed on day 7 post-wounding and post-infection with *S. aureus*. The bacterial numbers were 5.19 × 10^4^ and 1.71 × 10^7^ CFU/g of tissue in the uninfected and infected control groups, respectively. Moreover, the bacterial load ranged from 1.48 to 9.59 × 10^6^ CFU/g of tissue in the OAPO- or TMP-SMX-treated groups, which was significantly lower than that of the infected control group (*p* < 0.01; Figure 6A).

Micro-anatomical changes were assessed by HE staining on day 7 post-infection. In the infected control group, the wound showed higher inflammatory cell infiltration than in the uninfected control group. Moreover, there was a high prevalence of hemorrhage and red blood cells in the wounds of the infected control group. However, there were few inflammatory and red blood cells in the wounds of the TMP-SMX- and OAPO-treated groups on day 7 post-infection with *S. aureus* (Figure 6B).

**Figure 7:**
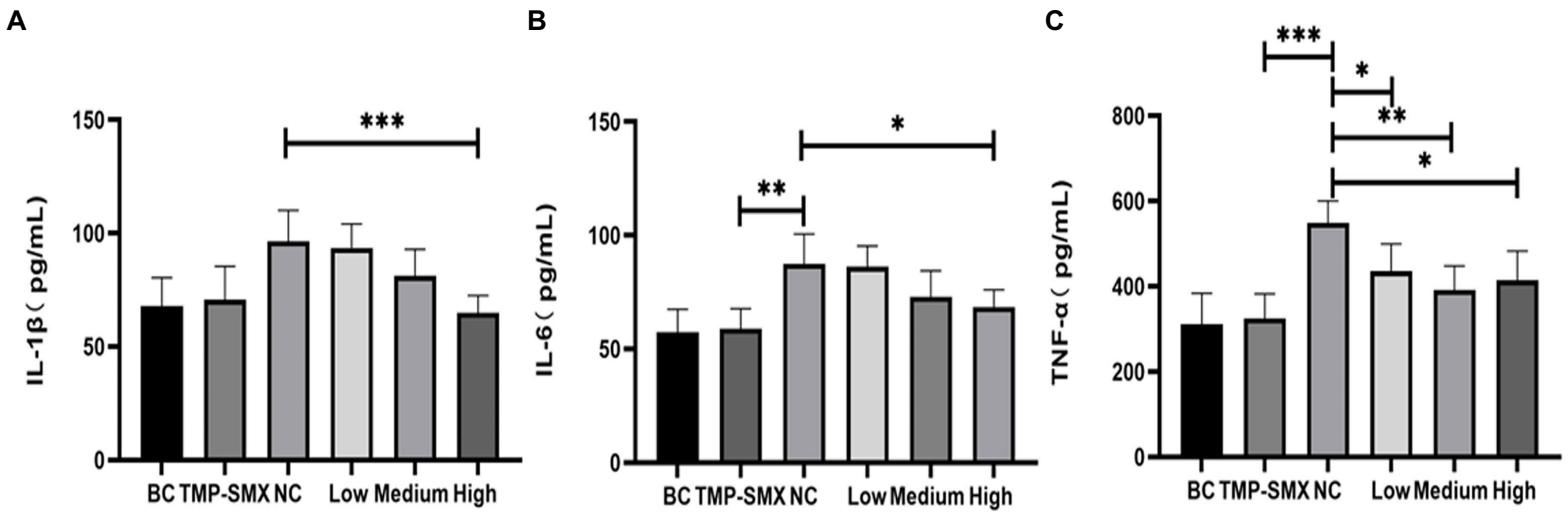
Determination of IL-1β **(A)**, IL-6 **(B)**, and TNF-α **(C)** in mice serum by enzyme-linked immunosorbent assay (ELISA). *indicates *p* < 0.05, **indicates *p* < 0.01, ***indicates *p* < 0.001 when compared with the negative control group (NC, untreated with organic acid of *Portulaca oleracea* L. [OAPO]). TMP-SMX: trimethoprim-sulfamethoxazole; BC: blank group, uninfected, no drug; NC: infected without the drug; low, medium, and high means groups were given a low, medium, and high dosage of OAPO, respectively.

Inflammatory responses were also investigated by measuring inflammatory cytokines in mouse serum (Figure 7). IL-1β, IL-6, and TNF-α levels significantly increased in mice in the infected control group. However, a high dose of OAPO decreased the levels of IL-1β and IL-6. Moreover, TNF-α levels were significantly decreased by both OAPO and TMP-SMX (*p* < 0.01).

## 4. Discussion

*Staphylococcus aureus* is an important zoonotic pathogen that not only causes local and systemic infections in humans but also induces dermatitis, mastitis, enteritis, and other infections in livestock and poultry (Zhou et al., 2018). The occurrence of methicillin-resistant or multidrug-resistant *S. aureus* strains makes the treatment of staphylococcal diseases more difficult. Moreover, the implementation of strict laws against antibiotic use in animals has complicated the husbandry of *Staphylococcus*-infected animals. However, many new methods could be applied to reduce antibiotic usage and kill *S. aureus*, such as herbal extracts, bacteriophages, acidifiers, and antimicrobial peptides. Aqueous extracts of *P. oleracea* have a slight inhibitory effect on *E. coli* (Okuda et al., 2021). However, the bacteriostatic mechanisms and active ingredients of *P. oleracea* remain unclear. The present study aimed to demonstrate that OAPO is the main active ingredient of *P. oleracea* against *S. aureus* through *in vitro* and *in vivo* experiments.

There have been many studies on the antimicrobial activity of *P. oleracea* against a broad spectrum of bacterial species (Soliman et al., 2017); however, the characteristics of antimicrobial activity determined by these studies were slightly different from those in ours. Tleubayeva et al. showed that the CO_2_ extract of *P. oleracea* had the greatest bactericidal effectiveness against *S. aureus* at 250 μg/ml and against *Escherichia coli*, *Bacillus subtilis*, and *Candida albicans* at 500 μg/ml (Tleubayeva et al., 2021). *Portulaca elatior* leaf lectin also showed strong activity against some phytopathogenic bacteria with a MIC value of 0.185 μg/ml (da Silva et al., 2021). The methanol extracts of *Portulaca quadrifida* at 250 μg/ml and 200 μg/ml showed the most significant antimicrobial activity against all tested gram-positive and gram-negative bacteria (Desta and Cherie, 2018). However, the present study showed that the MIC of OAPO was 12.5 mg/ml, which is inconsistent with the results of the above studies. The sources of herbal medicine and the tested bacterial strains were very different, and these factors might be the main causes of the different MIC results. Moreover, some compounds identified from *P. oleracea* exhibited only weak dose-dependent inhibition against bacterial pathogens, whereas other compounds showed significant antibacterial activity against common enteropathogenic bacteria *in vitro* (Lei et al., 2015; Hu et al., 2021). Interestingly, two unsaturated fatty acids from *P. oleracea* acted synergistically with erythromycin *in vitro* against MRSA, possibly by inhibiting the multidrug efflux pump of the bacteria (Chan et al., 2015; Fung et al., 2017). In summary, the specific substances and/or chemicals that play an antibacterial role in *P. oleracea* are still unclear.

*Portulaca oleracea* has many constituents, including flavonoids, alkaloids, organic acids, terpenoids, polysaccharides, vitamins, sterols, and other compounds (Zhou et al., 2015). In our preliminary tests, the aqueous extract, flavonoids and polysaccharide extracts did not display antibacterial activity (data not shown), while the organic acids possessed certain *Staphylococcus*-killing properties. Organic acid extracts were prepared according to a previously reported method, with slight modifications (Zhang et al., 2020). HPLC-MS was used to analyze the chemical composition of OAPO, and many organic acids were identified in OAPO, such as α-eleostearic, palmitic, phloionolic, and stearidonic acids. Some organic acids, such as protocatechuic and caffeic acid, were not identified in OAPO by HPLC-MS. There are two important reasons for this: one is that *P. oleracea* contains too little protocatechuic and caffeic acids to be detected, and the other is that the mixture of OAPO affects the measurement accuracy of HPLC-MS. Nevertheless, an organic acid-enriched extract was obtained in this study, which proved that OAPO could kill *S. aureus*.

The integrity of the bacterial cell wall and membrane is vital for survival. Many antibiotics can inhibit bacterial growth and induce loss of cell homeostasis by altering or damaging the cell membrane structure (Turgis et al., 2009). Different degrees of OAPO-induced damage were observed in *S. aureus* using SEM, and the lysis effects of OAPO on the cell membrane were dose-dependent, which indicated that this damage in the cell wall and membrane was likely responsible for the observed growth inhibition and bactericidal effects. To further demonstrate the extent of OAPO-induced cell wall damage, the presence of AKP and nucleic acid substances in the supernatant of OAPO-treated *S. aureus* culture medium was used as an indicator of cell wall integrity. AKP is an enzyme located between the bacterial cell wall and cell membrane and, therefore, is undetectable when the bacterial structure is intact. Nevertheless, when the cell wall is damaged, AKP leaks out of the cell owing to an increased permeability (Tang et al., 2017). The present study showed that OAPO was more effective at elevating extracellular AKP levels at higher concentrations, which indicated that OAPO could increase the permeability of the cell wall, and damage to the integrity of the cell wall might be the main cause of AKP release into the supernatant. Moreover, nucleic acids are essential life substances in microorganisms and cannot permeate through the cell membrane during the normal growth of bacteria (Wu et al., 2016). The degree of leakage of nucleic acids from *S. aureus* cells treated with one-fold MIC and two-fold MIC of OAPO was significantly higher than that from untreated *S. aureus*, which suggested that OAPO might induce outer membrane damage in *S. aureus*, causing nucleic acids to leak out from the bacterial cells. Our results are consistent with those regarding the antibacterial activity of lactobionic acid against *S. aureus* by breaking down the structure of the bacterial cell wall and membrane (Cao et al., 2019).

Protein synthesis and energy metabolism are two other factors that affect bacterial survival. Soluble proteins are important osmotic pressure regulators of bacteria, which can improve the water retention capacity of bacteria and ensure their normal life activities (Nguyen et al., 2019). In this study, we determined the prevalence of intracellular biomacromolecules, such as total protein, ATPase, and glucose-6-phosphate dehydrogenase, in *S. aureus* treated with OAPO. The results of SDS-PAGE and BCA assays showed that the soluble protein levels of *S. aureus* were significantly elevated by OAPO, which indicated that interference with protein metabolism might not be the antibacterial mechanism of OAPO. Furthermore, OAPO did not affect intracellular ATPase and glucose-6-phosphate dehydrogenase content (data not shown), thereby indicating that OAPO could not inhibit ATPase activity and could not prevent ion transportation and nutrient absorption (Petrosyan et al., 2015).

To verify that OAPO has the same *S. aureus*-killing effects *in vivo* as it did *in vitro*, an animal model of wound infection with *S. aureus* mimicking clinical conditions was used in our study. The skin is the first line of defense against diseases, and skin wound infection is a useful animal model of *S. aureus* infection, characterized by a series of pathological changes, from local to systemic infection (Edwards and Harding, 2004; Gupta and Tyagi, 2021). Therefore, we used a skin wound infection model induced by *S. aureus* to evaluate the anti-infective effects of OAPO *in vivo*. The results demonstrate three aspects of the antibacterial activity of OAPO. First, OAPO could significantly promote skin wound healing compared with that by TMP-SMX; therefore, the skin histopathology of mice in the OAPO group was close to that of normal skin. It has long been reported that the crude extract of *P. oleracea* accelerates the wound healing process by decreasing the wound surface area and improving tensile strength (Rashed et al., 2003). Moreover, OAPO promotes wound healing better than homogenized plant material, indicating that organic acids might be the main active ingredients for improving wound injury. Second, the bacterial skin load in the OAPO group was significantly lower than that in the self-healing group, especially the bacterial load of the low-dosage group, which was significantly lower than that of the TMP-SMX group. Chan et al. reported that two fatty acids from *P. oleracea* exhibited synergistic effects with erythromycin in combating MRSA (Chan et al., 2015). Other organic acids from *P. oleracea*, such as protocatechuic and palmitic acids, also displayed effective bactericidal activity, which was in accordance with our results (Chao and Yin, 2009; Song et al., 2020). Third, the inflammatory responses induced by *S. aureus* were used to evaluate the anti-inflammatory effects of OAPO. The results demonstrated that OAPO exerted inhibitory effects on inflammatory cytokine expression, which was consistent with the results of previous studies on the anti-inflammatory activity of *P. oleracea* extracts (Rahimi et al., 2019; Moslemi et al., 2021).

## 5. Conclusion

To conclude, an extract of *Portulaca oleracea* was obtained in this study and it not only inhibits methicillin-resistant *S. aureus* activity *in vitro* but also inhibits *S. aureus*-induced skin damage in a mouse model. The chemical compositions of the extract contain lots of organic acids, which indicates that OAPO is the main active compositions against MRSA. However, the purity and toxicity of the extracted organic acids was unknown, and which organic acid has stronger antibacterial activity needs to be explored soon.

## Data availability statement

The original contributions presented in the study are included in the article/Supplementary material, further inquiries can be directed to the corresponding author.

## Ethics statement

The animal study was reviewed and approved by the Animal Management and Ethics Committee of Guangxi University.

## Author contributions

GL, HS, and CO conceived and designed this study. GL, AL, CZ, QZ, CY, HL, and HY participated in laboratory work. JM, ZZ, GL, and AL performed the data analysis and the writing of the manuscript. AL and CO participated in revising the manuscript critically. All authors contributed to the article and approved the submitted version.

## Funding

This work was supported by scientific research startup funds of Guangxi University. We would like to thank Editage (www.editage.cn) for English language editing, Guangxi Science and Technology Base and Talent Special (2021AC19372) and TCM Industrial Pioneers (GuiNongKeMeng202211).

## Conflict of interest

The authors declare that the research was conducted in the absence of any commercial or financial relationships that could be construed as a potential conflict of interest.

## Publisher’s note

All claims expressed in this article are solely those of the authors and do not necessarily represent those of their affiliated organizations, or those of the publisher, the editors and the reviewers. Any product that may be evaluated in this article, or claim that may be made by its manufacturer, is not guaranteed or endorsed by the publisher.
